# Substance withdrawal during psychotherapy incorporating equines: A preliminary investigation of the role of neurotransmitters during treatment

**DOI:** 10.1016/j.eqre.2025.100035

**Published:** 2025-07-01

**Authors:** M.M. Friend, M.C. Nicodemus, C.O. Lemley, C.A. Cavinder, P. Prince, K. Holtcamp

**Affiliations:** a Huck Institutes of Life Sciences, The Pennsylvania State University, 101 Huck Life Sciences Building, University Park, PA 16802, USA; b Department of Animal and Dairy Sciences, Mississippi State University, Box 9815, MS 39762, USA; c Office of Psychological Services, College of Veterinary Medicine, Mississippi State University, PO Box 6100, MS 39762, USA

**Keywords:** Addiction, Substance abuse disorder, Human-animal interaction, Psychotherapy incorporating equines, Neurotransmitters

## Abstract

Substance withdrawal presents a barrier to substance use disorder (SUD) recovery in part due to symptoms implicating neurotransmitters and their metabolites. Despite this relationship, research investigating alternative treatments such as psychotherapy incorporating equines (PIE) have primarily targeted vital signs and cortisol concentrations. Previous research has also proposed benefits of human-horse physiological coupling, but the role of neurotransmitters in the relationship between humans and horses during PIE has not yet been investigated. The purpose of this study was to characterize neurotransmitter concentrations of humans and horses throughout PIE in withdrawing SUD patients and determine neurotransmitter involvement in human-horse physiological coupling. Saliva samples were collected from humans (n = 6) and horses (n = 4) immediately prior to and following PIE sessions in the first and second week of SUD treatment. Concentrations of 3-methoxytyramine (3-MT) in the second week of treatment increased (*P* = 0.04) in human participants, and concentrations of serotonin (5-HT) and Homovanillic acid (HVA) decreased (*P* = 0.04; *P* = 0.03), which may be indicative of withdrawal symptoms improvement. Horses exhibited increased concentrations of 5-HT (*P* = 0.02) and decreased concentrations of 3-MT (*P* = 0.01), which could reflect positive interactions within PIE. Patients and horses also developed correlations in 5-HT (0.99, *P* = 0.05), DA (0.20, *P* < 0.01), 3,4-dihydroxy-phenyl acetic acid (DOPAC) (0.70, *P* = 0.01), and glutamate (0.66, *P* = 0.02) concentrations over the course of PIE sessions, indicating human-horse coupling associated and providing support of physiological synchronization between humans and horses participating in PIE.

## 1. Introduction

Substance use disorder (SUD) is prevalent within the United States, claiming over 700 thousand lives in the past two decades [1]. The Diagnostic and Statistical Manual of Mental Disorders, Fifth Edition, (DSM-5) defines SUD as a range of compulsive drug use patterns despite negative consequences and characterized by cognitive, behavioral, and physiological symptoms. This condition encompasses multiple classes of drugs including alcohol, cannabis, hallucinogens, inhalants, opioids, sedatives, hypnotics, stimulants, and tobacco [2]. The physical and cognitive challenges related to substance withdrawal symptoms may be a driving force to reoccurring treatment failure for SUD patients, as 30–50 % of patients discharge against medical advice in the first three months of treatment [3,4]. Withdrawal is defined as the period in which circulating and tissue concentrations of the substance decline following persistent use. Symptoms of withdrawal vary widely between substance classes, but the state of withdrawal is associated with symptoms initiated by cessation of use [2]. Past studies have demonstrated imbalances in neurotransmitters and glucocorticoids during withdrawal associated with detrimental symptoms such as craving, fatigue, depression, agitation, and diminished cognition [5–8]. These symptoms are largely controlled within the medial prefrontal cortex, an area of the brain severely affected by addiction and the terminal location of dopamine (DA) [9,10].

### 1.1. Neurotransmitters in withdrawal

Dopamine is a neurotransmitter, which is a chemical messenger that transmits signals from nerve cells to target cells [11]. The production and pathway of this neurotransmitter is important in the control of addiction [12,13]. Many addictive drug classes increase DA concentrations in the brain, and with continuous use, DA receptors eventually become desensitized [9]. Concentrations of other neurotransmitters such as serotonin (5-HT) and gamma-aminobutyric acid (GABA) can be increased by drug use, which can also further impact concentrations of DA and contribute to bidirectional dysregulation of neurotransmitters [14].

The dysregulation of the synthesis and metabolism of these neurotransmitters play an important role in substance withdrawal symptoms, and therefore impact recovery [15]. As such, it is important to determine the impacts of treatment on concentrations of neurotransmitters involved in addictive pathways, memory, and motivation in order to develop therapies that more effectively address symptoms that create barriers to recovery. Understanding changes in concentrations of neurotransmitter metabolites such as dihydroxyphenylalanine (DOPA), 3-methoxytyramine (3-MT), Homovanillic acid (HVA), and 5-Hydroxyindoleacetic acid (5-HIAA) contributes insight into how treatment methods and withdrawal progression affects neurotransmitter dysregulation seen in addiction and withdrawal.

Measuring neurotransmitter concentrations has traditionally been relatively invasive, with cerebral spinal fluid serving as the gold standard of measuring central neurotransmitter concentrations. While neurotransmitters are not able to cross the blood-brain barrier, multiple studies have shown peripheral concentrations of neurotransmitters, such as GABA, glutamate, and DA are altered in addiction and cessation of drug use [16,17]. Additionally, metabolites such as HVA and dopamine sulfate can cross the blood-brain barrier and appear at altered concentrations in peripheral circulation during addiction and withdrawal from certain drugs [18,19]. Measures of peripheral concentrations of neurotransmitters, such as in the blood or saliva, present the opportunity for less invasive means of measuring neurotransmitter concentrations to understand physiological effects of substance abuse, withdrawal, and treatment.

### 1.2. Impacts of human-equine interaction during PIE

Psychotherapy incorporating equines (PIE) has grown as an alternative treatment option, yet the mechanism of these benefits has yet to be elucidated [20]. This therapeutic intervention is an experiential-type psychotherapy in which the horse is incorporated into therapeutic sessions while patients are treated by a licensed psychotherapist in the equine environment. Past studies have demonstrated the ability of PIE to decrease cortisol concentrations [21], as well as improve retention rates [22], therapeutic alliances [23], and communication skills [24]. These results hold promise for the use of PIE in withdrawing SUD populations, particularly as it pertains to symptoms associated with neurotransmitter dysregulation.

Recent research has considered potential physiological coupling between humans and horses during treatment. Friend et al. [20] reported negative correlations between human and equine cortisol concentrations in SUD treatment populations, as human cortisol concentrations decreased as horses’ increased, a change attributed to increased activity due to diminished withdrawal-associated pain. Alternatively, Holtcamp et al. [25] documented positive correlations between young adults with SUD and equine respiratory rates, heart rates, and pain scales during PIE-supplemented collegiate recovery community programming. A study investigating the use of PIE in treatment of children diagnosed with post-traumatic stress disorder found correlations between human and equine cortisol concentrations occurring within six days of PIE, and human-equine interaction outside of PIE have demonstrated correlations in heart rate variability [26–30].

Despite work towards understanding physiological coupling, neurotransmitter concentrations have scarcely been studied in PIE-type interactions. The sole report found that horses exhibited smaller increases in plasma epinephrine when interacting with children with autism spectrum disorder compared to neurotypical children [31]. However, no further studies are currently available examining neurotransmitter responses within human or equine participants, pointing to a notable gap in the literature of this field. Additionally, neither salivary concentrations of neurotransmitters in horses nor correlations between human and horse neurotransmitters have been studied. Salivary neurotransmitter concentrations have been measured using liquid chromatography-mass spectrometry (LCMS) [32–34]. This means of analysis has been used in humans but not yet horses. The inability to include validation for horses in the scope of this study presents a common limitation, but results of this preliminary, proof-of-concept study can serve as an initial step towards future work and validation in the field.

### 1.3. Objectives and aims

Research investigating physiological benefits of PIE in withdrawing SUD patients is limited but can have a significant impact on the evaluation of the efficacy of this alternative therapeutic intervention. Studies are specifically lacking concerning the impact of PIE on neurotransmitters in SUD humans and their equine counterparts. Thus, the objectives of this study were twofold: 1) to characterize concentrations of a panel of neurotransmitters and their metabolites in the saliva of withdrawing residential SUD patients and their equine counterparts and 2) to determine physiological coupling of the neurotransmitters between humans and horses during the treatment process.

## 2. Materials and methods

All procedures in this study were approved by Mississippi State University Institutional Review Board (IRB) protocol # 22–482 and Institutional Animal Care and Use Committee (IACUC) protocol # 21–306.

### 2.1. Treatment facility and program

The present study was conducted at the American Addiction Centers’ Oxford Outpatient Clinic in Oxford, MS. The facility operates partial hospitalization and intensive outpatient programs that include individuals with minimal sober time with continued withdrawal symptoms. Patients in this program participated in daily psychotherapy sessions aimed at substance abuse recovery. PIE sessions were conducted by an accredited mental health professional with licensure and experience in PIE. Once per week, the therapist conducted PIE at the treatment facility utilizing a program that had been developed for and previously utilized within the recovering SUD population. Data collection took place between June 1 and July 27, 2022. The sessions lasted an average of 21 min, beginning at 0830 h. Each session consisted of ground-based activities which utilized the horse in activities intended to prompt therapeutic conversations related to treatment goals as previously outlined in Friend et al. [20]. The equine therapist systematically performed these session themes and activities each week in the order of the provided table (Table S1). Participants worked with a different horse for each session of this study.

### 2.2. Human participants

Participants within this study (n = 6) were withdrawing SUD patients during the first two weeks of PIE (Table 1). Study participants were recruited by the treatment facility. Study inclusion was open to all patients enrolled in the treatment program and those willing to participate in PIE activities. Patients who were discharged before six weeks of treatment were removed from the study. Patient participation was voluntary and did not affect patients’ ability to participate in any aspect of treatment. Participants maintained the right to cease treatment and/or study participation at any time. The average age of study participants was 34 ± 14.8 years old. Participants stayed at the treatment facility an average of 69 ± 63 days and participated in standard group and individual treatments outside of PIE during that time. While drug use prior to enrollment in the treatment program varied among participants, the most common drug of choice according to the mental health professionals coordinating treatment included opioids and methamphetamine (Table 1). The mental health professionals reported no participants had prior equine experience. Additional information regarding patient comorbidities, medication, and other treatment was unavailable due to confidentiality restrictions from the treatment facility.

### 2.3. Horse participants

Horses (n = 4) used in PIE for this study were experienced in working with the SUD population. Horses were on average 14 ± 3.5 years old and represented multiple horse breeds (Table 2). All horses had been used for SUD treatment programs for at least six months at the facility and specifically within the program that was utilized for this study. Prior to the study, horses were familiarized with researchers and data collection methods as discussed in the following section. Horses were group-housed in pastures for the duration of this study and fed a consistent diet composed of commercially available forage and pelleted concentrate with constant access to clean water. Management practices remained consistent throughout the trial. The health of horses during this study was monitored by the Mississippi State University College of Veterinary Medicine. The veterinarians did not report any health issues that would impact the data collected prior to the study and reported no changes in horses’ health status during the study. During the study, horses’ use outside of activities associated with the study included on average zero to one hour of work per week consisting of riding lessons and/or PIE sessions at the horses’ home facility.

#### 2.3.1. Saliva sample collection

Saliva samples were collected from both humans and horses using Salimetrics adult and children’s swabs, respectively (Salimetrics, State College, PA, USA). Human and equine samples were taken within five minutes prior to (pre) and following (post) PIE treatment in the first (W1) and second (W2) weeks of treatment. Samples were immediately stored atop dry ice then stored at −80° C within six hours of collection.

### 2.4. Neurotransmitter panel analysis

Human and equine saliva samples were shipped to Vanderbilt University Neurochemistry Core (Nashville, Tennessee) operated by the Vanderbilt Brain Institute and Kennedy Center for Research on Human Development. Samples were sent in two batches (the first batch contained n = 3 first-week human samples and n = 2 equine samples; the second batch contained n = 3 first-week human samples, n = 3 first- and second-week human samples and n = 2 first- and second-week equine samples). The batch of samples was accounted for in the statistical analysis.

Laboratory procedural guidelines for sample analysis followed that reported by Song et al. [35]. Aliquots of 250 μL of human (n = 6) and equine (n = 4) saliva samples were analyzed using LCMS. Stock solutions of the biogenic amines analyzed (5 ng/μL each) were made using deionized water. To create internal standards, stock solutions were derivatived using isotopically labeled benzoyl chloride. 50 μL of the stock solution was diluted with 200 μL acetonitrile. 100 μL of both 500 mM NaCO3 (aq) and 2 % 13C6-BZC in acetonitrile was added to the solution and allowed to react for two minutes. After two minutes, the reaction was stopped with the addition of 200 μL of receptor 1 A (HTR1A), solute carrier family 6 member 4 (SLC6A4), monoamine oxidase A (MAOA), and tyrosine 3-monooxygenase/tryptophan 5-mono-oxygenase activation protein beta (YWHAB).

Biogenic amines detected were dopamine (DA), homovanillic acid (HVA), 5-hydroxyindolacetic acid (5-HIAA), serotonin (5-HT), 3,4-dihydroxy-phenyl acetic acid (DOPAC), glutamate (Glut), Gamma-aminobutyric acid (GABA), 3-Methoxytyramine (3-MT), arginine (Arg), taurine, and lysine (Lys). The concentrations are denoted in pg/μl of saliva. DA and 5-HT for humans (n = 3) and horses (n = 3) were only analyzed in the first week of PIE due to updates within the neurotransmitter panel analytics made by the laboratory.

### 2.5. Statistical analysis

Data were tested for normality using the Shapiro-Wilks test of SAS version 9.4 (SAS Institute Inc., Cary, NC, USA). Data found to be nonparametric were log transformed when possible. When transformed data remained nonparametric, data were ranked using the Wilcoxon Signed Rank test. Data were analyzed using the MIXED procedure with fixed effects of week, treatment, week by treatment interaction, drug of choice, and sample batch. A repeated measures statement was used on parametric and normalized data. When interactions were *P* > 0.10, they were removed from the model. Drug of choice and sample batch were removed from the model in a backwards stepwise manner if *P* > 0.20. Means were separated using the PDIFF option within the LSMEANS statement. Correlations were evaluated using the Spearman function of the CORR procedure to compare human and horse neurotransmitter concentrations. Statistical significance was declared at *P* ≤ 0.05. Means and standard deviations were reported.

## 3. Results

### 3.1. Human neurotransmitters

**Fig. 1.**
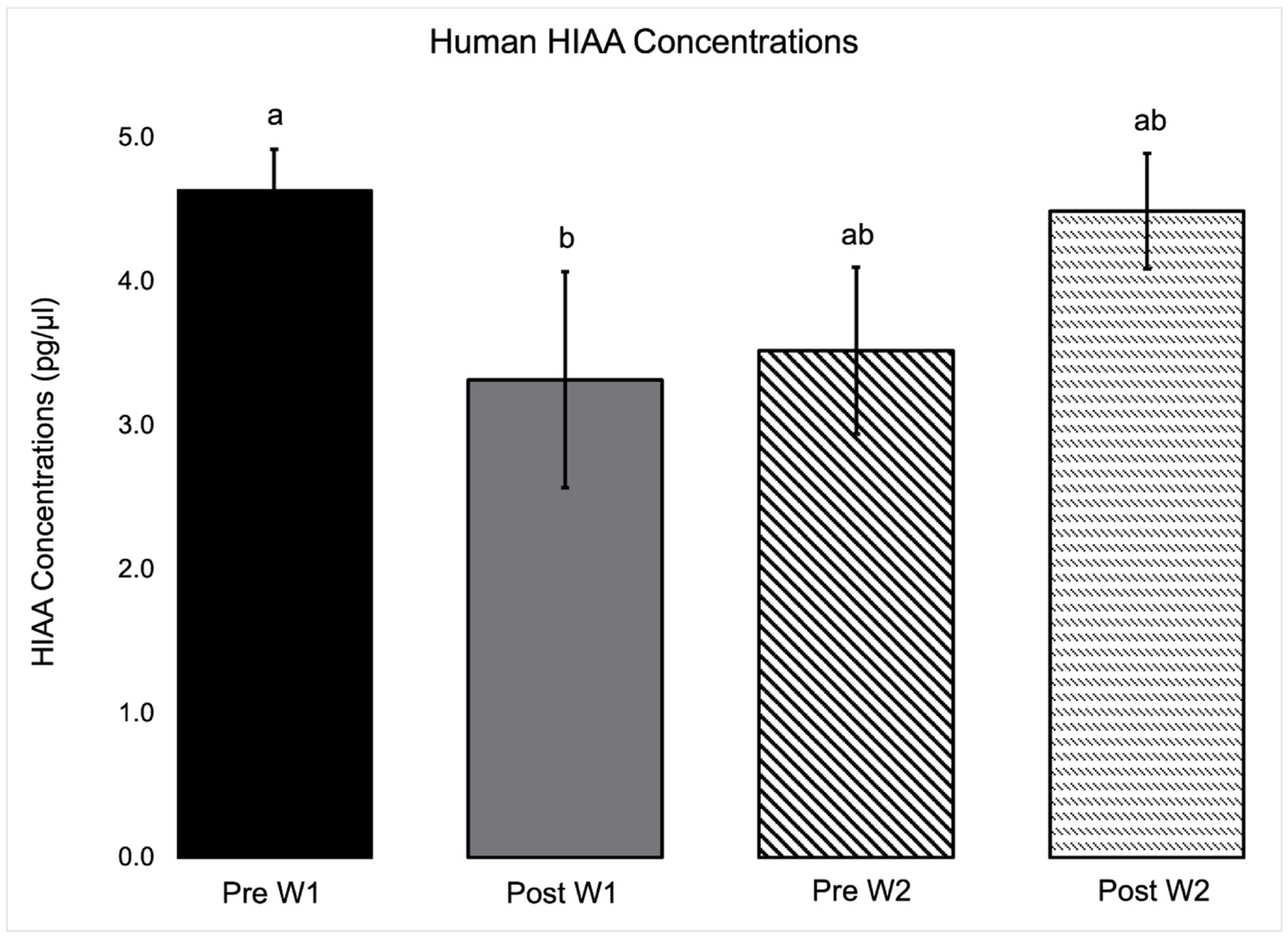
Human 5-HIAA concentrations were lower post W1 session of PIE during a substance abuse disorder residential treatment program. Different subscripts between bars indicating significant difference (P ≤ 0.05).

Human neurotransmitter concentrations from saliva samples are reported in Table 3. A main effect of week was found in HVA (*P* = 0.03) and GABA (*P* < 0.01) with concentrations of HVA and GABA lower in patients throughout W2, both pre and post treatment. Alternatively, there was a main effect of week in which 3-MT (*P* = 0.04) was higher in patients throughout W2 both before and after PIE sessions. Both 5-HT (*P* = 0.04) and 3-MT (*P* = 0.01) showed a main effect of treatment with concentrations lower post treatment. A week by treatment interaction was observed in 5-HIAA (*P* = 0.01) concentrations, with concentrations post W1 session being lower than pre W1 (Fig. 1).

### 3.2. Horse neurotransmitters

**Fig. 2.**
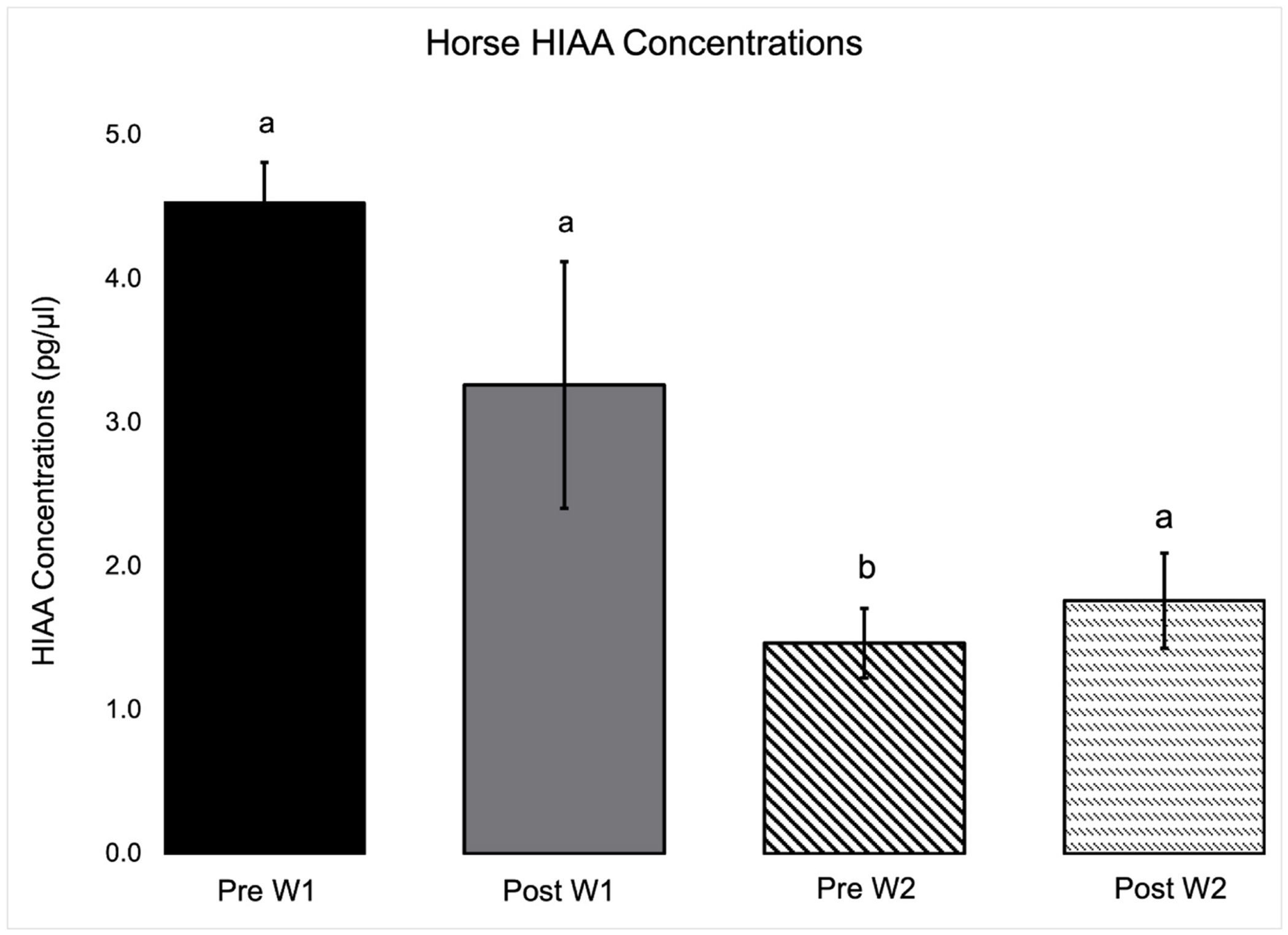
Horse 5-HIAA concentrations were lower Pre W2 of PIE during a substance abuse disorder residential treatment program. Different subscripts between bars indicating significant difference (P ≤ 0.05).

Horse neurotransmitter concentrations from saliva samples are presented in Table 4. Main effects of week were found in the concentrations of 3-MT (*P* = 0.03), lysine (*P* = 0.01), and taurine (*P* < 0.01). Concentrations of lysine and taurine were higher pre and post W2, while 3-MT concentrations were lower pre and post W2. A main effect of treatment was found for 5-HT, 3-MT, and taurine in which concentrations of 5-HT (*P* = 0.02) and taurine (*P* = 0.03) were higher post-treatment across weeks and concentrations of 3-MT (*P* = 0.01) were lower post-treatment across weeks. A week by treatment interaction was found in which 5-HIAA (*P* = 0.05) concentrations were lower pre W2 session as compared to all other time points (Fig. 2).

### 3.3. Human-horse neurotransmitter correlations

Correlations between human and horse neurotransmitter concentrations are reported in Tables 5 and 6. There was a moderate positive correlation (0.65, *P* = 0.02) between 5-HIAA in humans and horses pre-treatment across weeks. There were strong positive correlations between human and horse 5-HT concentrations (0.99, *P* = 0.05) and between human and horse DOPAC concentrations (0.70, *P* = 0.01) post-treatment across weeks. There was also a moderate positive correlation (0.66, *P* = 0.02) between human and horse glutamate concentrations post-treatment. Human and horse DA concentrations showed a weak positive correlation (0.20, *P* < 0.01) post-treatment across weeks as well.

## 4. Discussion

No studies prior to the current study have considered the impact of PIE on salivary neurotransmitter concentrations in SUD patients; yet, an understanding of salivary neurotransmitter profiles of withdrawing SUD patients may be valuable in evaluating physiological impacts of treatments due to the non-invasive nature of sample collection. This is the first study reporting salivary biogenic amine and neurotransmitter profiles in horses and in SUD withdrawing humans in treatment.

### 4.1. Neurotransmitter concentration changes in humans

Decreased concentrations of salivary 5-HT in patients following treatment were unexpected, as 5-HT is often linked with pro-social behavior [36]. However, this neurotransmitter also plays an important role in pain sensitivity [36,37]. Peripheral 5-HT can act to sensitize nerve fibers in the periphery to increase sensitivity to pain but can have analgesic effects in the central nervous system [38]. Because of these important roles in pain sensation, 5-HT is released from platelets and mast cells at the location of tissue injury, inflammation, or painful stimuli [39]. Substance withdrawal is well-documented as a physically uncomfortable and often painful experience, consisting of symptoms such as muscle pain, fatigue, headaches, and even seizures [40–42]. Analgesic effects of exercise are well-documented, and it is possible the physical activity involved in PIE may have positive impacts on withdrawal patients’ pain [43,44]. Elevated concentrations of plasma 5-HT and its metabolite 5-HIAA have been documented in individuals with depression [45]. Reduced concentrations may therefore be indicative of improvements in this condition associated with withdrawal [46,47]. When considered together, this information suggests further research should be conducted to determine if patients participating in PIE experience improvements in symptoms of withdrawal, perhaps in part due to the introduction of physical activity, resulting in lower 5-HT release. This is supported by the results of a study investigating the impacts of PIE on SUD populations’ vital signs and pain scales, in which authors found SUD patients’ pain scales decreased over the course of a PIE session [25].

While 5-HT was only measured in the first week of treatment, the week by treatment effect of PIE on 5-HT metabolite 5-HIAA offers further insight into continued changes in the second week of treatment. It is suspected low 5-HT concentrations following the first week of treatment were reflected in reduced 5-HIAA concentrations. This change in 5-HIAA was not seen in the second week of treatment, and we therefore suspect 5-HT decreased following treatment in the first week but remained stable throughout the second week of treatment. The interaction may indicate patients experienced less variability in pain prior to and throughout treatment in the second week of treatment, as acute withdrawal symptoms began to resolve and patients became less apprehensive about the treatment and working with horses.

As expected, patients in this study exhibited neurotransmitter concentrations indicative of substance withdrawal, particularly in the second week of treatment with decreased GABA. Low concentrations of this neurotransmitter, even in plasma, are associated with withdrawal and symptoms such as hallucination and difficulty concentrating [16]. Thus, while changes in 5-HT concentrations in the second week may indicate diminished physical symptoms, some psychological and cognitive symptoms associated with GABA dysregulation may persist [15]. Decreased concentrations of dopamine metabolite HVA were also seen. Past studies have found positive correlations with plasma HVA and substance craving, so patients may have experienced reductions in associated withdrawal symptoms [48,49]. However, increased concentrations of 3-MT, a DA metabolite preceding HVA, were seen in the second week of treatment. Many drugs upregulate DA release, and thus elevate the subsequent metabolite, 3-MT. While central DA is responsible for addictive pathways, changes can be seen in peripheral DA metabolites in withdrawal as well [18]. The metabolite 3-MT is formed through the activity of catechol-o-methyltransferase (COMT), and monoamine oxidase (MAO) subsequently breaks 3-MT down into HVA. The increase in 3-MT but decrease in HVA may therefore indicate a suppression of MAO even in the second week of treatment. This finding is reasonable, as past research has found a correlation between drug dependence and peripheral COMT mRNA [50]. It is possible the increase in DA synthesis and beginnings of metabolism exemplified by increases in 3-MT indicate dopaminergic dysregulation stabilization by the second week of treatment, though damage to MAO activity due to substance abuse persisted [51,52]. Alternatively, it is important to consider the timeline of peripheral, specifically salivary, neurotransmitter concentration changes, as they may vary between neurotransmitters or not be as rapid as central concentrations. While these explanations are purely speculative in the context of this study, these possibilities present an avenue for future research into the measurement of salivary neurotransmitters.

The decrease in 3-MT concentrations following treatment compared to the increase seen in the second week of treatment likely reflects differences between the effect of time in PIE treatment over the course of weeks and acute PIE treatment effects throughout sessions. This change was seen over the course of PIE sessions in both weeks of treatment, so the decrease in 3-MT concentrations following treatment can be most reasonably attributed to activities and interactions encompassed within treatment sessions. In this context, the role of DA in anticipation may be important, as central DA signaling is integral to motivation and reward-seeking behavior and peripheral DA is elevated in psychologically stressful circumstances and anticipation [53,54]. Thus, 3-MT concentrations may indicate this upregulation of DA. However, it again must be considered that the degree, rate, and duration of variation of DA concentration changes in the periphery and specifically in saliva may be different than what has previously been seen in central DA. However, throughout the treatment process, it is suspected DA concentrations would fluctuate as patients experienced successes and positive feedback as well as difficult topics and conversations throughout the session. As sessions ended, patients would no longer experience the same feelings of anticipation or stress, leading to comparatively lower DA and 3-MT concentrations. The fluctuation of DA throughout PIE sessions requires further research, but this finding could be encouraging to the impact of PIE in influencing positive learning experiences in SUD treatment.

### 4.2. Neurotransmitter concentration changes in horses

There is a lack of research surrounding neurotransmitter concentrations in horses used for PIE yet understanding horses’ responses to therapeutic use is important to further understanding the potential for human-horse physiological coupling in treatment. The changes in neurotransmitter concentrations in the second week of treatment can be most reasonably attributed to the horses’ reaction to changes in human patients due to the horses’ experience in PIE and the associated activities. Research has demonstrated horses are sensitive to human facial expressions, vocal tones, and chemo-signals, exhibiting increased heart rates in response [55–57]. It is therefore reasonable to speculate horses may have a negative response to individuals experiencing distressing symptoms of substance withdrawal. It is interesting that horses experienced decreases in 3-MT concentrations, indicating reduced DA turnover. It is suspected DA release decreased due to the absence of stress in addition to inhibition by 5-HT [58]. Past studies have proposed peripheral DA as a viable marker for fearfulness based on reports of less fearful horses exhibiting higher plasma DA concentrations and mice supplemented with DA precursor L-DOPA having improved tolerance for fearful stimuli [59–61].

Regarding changes in 5-HT concentrations, increases in 5-HT concentrations have been seen following exercise in horses [62–64]. While PIE does not require horses to participate in extensive exercise, a degree of movement greater than ordinary resting and grazing is involved. Regarding behavior, Kim and colleagues [60] discovered the notable absence of a relationship between plasma 5-HT and fearfulness and a positive association between 5-HT and low-dominance temperaments. The authors speculated 5-HT may play a role in cooperation, trust, and trainability in horses. Applying these interpretations to the present study, PIE-participating horses may have exhibited more cooperative and trusting behaviors throughout the PIE sessions. The activity of 5-HIAA may also be important in inferring activity of 5-HT across the weeks of treatment, which could not be done in this study due to laboratory changes. Contrary to expectations, decreased concentrations of 5-HIAA were seen prior to the second week of treatment, and raw means of plasma 5-HIAA in horses were lower in the second week, though it was not a statistically significant main effect in this small study population. Future studies should investigate this further, as past research has demonstrated that horses exhibit lower concentrations of stress hormones and neurotransmitters during interactions with populations with no equine experience or individuals on the autism spectrum undergoing PIE treatment [31,63]. Therefore, it is possible that horses could respond to patients’ increasing experience and comfort around the horses, which potentially led to more commands and higher expectations during the interaction. This could lead to an overall decrease in 5-HIAA and 5-HT in response.

Horses exhibited increased concentrations of lysine and taurine in the second week of treatment. Lysine is a necessary precursor of de novo glutamate synthesis, a neurotransmitter with neurons that synapse onto DA neurons with inhibitory effects [65]. This may help explain decreases in 3-MT also seen in the second week of treatment. Glutamate has many roles as a primary excitatory neurotransmitter, and it is important in attention, learning, and spatial training [66,67]. As such the increase in this amino acid may be indicative of increasing awareness of patients’ movements and receptiveness to commands. Taurine is a GABA receptor agonist, and GABA is similarly involved in motor learning, seeming to further support this explanation [68]. Taurine can be affected by activity as well. Even moderate levels of exercise can increase for muscle repair and increase antioxidative activity [68–70]. This may be responsible for a degree of elevation throughout treatment, alongside the potential causes previously discussed.

### 4.3. Neurotransmitter correlations between humans and horses

An interesting finding of this study is the development of correlations between human and horse neurotransmitter concentrations over the course of PIE. Prior to PIE sessions, a moderate positive correlation between 5-HIAA was found between humans and horses. This indicates 5-HT turnover and may indicate positive anticipatory emotions or response to the environment preceding the session. For human participants, this entailed time outside working with other patients and therapists before equine interaction. For horses, this entailed grazing without human interaction. After treatment, strong positive correlations between humans’ and horses’ concentrations of 5-HT and DOPAC emerged along with a moderate positive correlation of glutamate and a weak positive correlation of DA. The development of the 5-HT correlations may speak to the development of a social sensitivity between humans and horses during PIE or environmental response, as 5-HT is involved in the awareness of social factors and modulation of social responses [36]. The development of DOPAC and DA correlations suggest a synchronized anticipatory and motivational state between humans and horses participating in PIE [51,52]. This is strengthened by the synchronization of glutamate, which is an excitatory neurotransmitter important for facilitation of memory and cognition [65]. This largely agrees with past studies investigating the physiological relationship between humans and horses in therapeutic settings. A study conducted by Yorke and colleagues in 2012 found mild positive correlation in cortisol concentrations between children undergoing PIE treatment for post-traumatic stress disorder and their equine counterparts after six days [26]. Another study investigating the use of horses in an assisted living facility found 16 out of 24 residents experienced matching heart rate variability oscillation frequencies [30]. As for the SUD population, one study reports a moderate positive correlation between humans’ and horses’ respiration rates [71] while another study found a strong negative correlation between humans’ and horses’ salivary cortisol concentrations over the course of PIE sessions [20]. The results of this study add further evidence to the expanding body of literature documenting correlations indicative of physiological coupling between horses and patients in PIE treatment.

### 4.4. Study limitations

Although this study is the first to investigate the impact of PIE on a panel of salivary biogenic amines and neurotransmitters in human and horses participating in this therapeutic intervention, the novelty of the study resulted in limitations for future researchers to address. To the authors’ knowledge, this study was the first to quantify neurotransmitters in equine saliva despite the growing value of salivary samples in research [72]. Past studies have validated LCMS methods to determine concentrations of peripheral neurotransmitters in humans, but future studies should aim to validate these methods in the horse and individuals undergoing substance withdrawal, as this study was unable to do so [32,33]. Measurement of plasma neurotransmitter concentrations was not possible due to the invasiveness of blood sample collection in this population and scope of this study. Future research should work to investigate the relationship between salivary and plasma concentrations of neurotransmitters for more robust result interpretation. While both these factors present important limitations of this study that should be considered in interpretation of results, they are common limitations as salivary neurotransmitter analysis grows in popularity [34].

A primary limitation of this study is the sample size. While this was a preliminary investigation and the first to document neurotransmitter responses to PIE, further studies should consider expanding sample sizes to investigate neurotransmitters of interest identified in this study. This may allow for a more diverse population to study. Drug of choice was included as a covariate in models for HVA, HIAA, 5-HT, GABA, and was a significant predictor in HVA concentrations. Controlling the variation drug of choice of patients may benefit future studies as this could not be accomplished due to limited sample size.

The length of investigation could be expanded in future studies. The abbreviated PIE treatment curriculum was selected for this study due to previous findings that identified improvements in residential SUD patient emotional safety and procedural memory development in as little as two weeks [73]. However, studies in the future may consider longer time frames to account for varying withdrawal periods of substances and individual variation [40,74]. Extension of investigation length, nonetheless, should incorporate information from a validated withdrawal scale or psychological evaluations to monitor withdrawal symptoms and progression. This information at this time was unavailable for the time period investigated and thus will be an additional hinderance when extending length of investigation without future research. Incorporation of these measures in the understanding of the relationship between physiological changes throughout withdrawal and the psychological implications will be of value for clinical applications.

This study encountered an unbalanced panel of neurotransmitter analysis due to changes in batch analysis from the laboratory. Both DA and 5-HT were only analyzed in W1 samples due to laboratory changes. Future studies may consider analyzing DA and 5-HT in both the first and second weeks rather than drawing inferences from metabolites. Future investigations may consider adding further analysis of biomarkers including hormones such as epinephrine, norepinephrine, and oxytocin, leading to a more complete understanding of humans’ and horses’ response to PIE. These biomarkers could identify stress responses, or alternatively, the development of bonds and attachment throughout interactions, and are beginning to be investigated throughout human-equine and PIE-type interactions [75,76]. Nonetheless, this study is the first to investigate neurotransmitters within PIE participants during the withdrawal process, and as such, previous work was unavailable to offer a foundation for which specific neurotransmitters to investigate.

## 5. Conclusions

This study presents the first concurrent examination of withdrawing SUD patient and equine neurotransmitter concentrations throughout the first two weeks of PIE in an effort to document neurotransmitter concentrations of both human and equine PIE participants and investigate the involvement of neurotransmitters in physiological human-horse coupling during treatment. Notably, SUD patients experienced increased DA metabolite 3-MT and decreased DA metabolite HVA in the second week of treatment, which may indicate differences in the recovery of the dopaminergic pathway in COMT and MAO pathways. Additionally, patients experienced decreased concentrations of 5-HT and HVA, which can indicate improvements in withdrawal symptoms, though dysregulation of other neurotransmitters such as GABA persisted. Horses experienced increased concentrations of 5-HT and decreased concentrations of 3-MT over the course of treatments, seeming to indicate positive interactions. Perhaps most notably, humans and horses developed correlations in salivary neurotransmitter responses over the course of treatment sessions. This seems to strengthen evidence of physiological coupling between humans and horses during PIE.

## Supplementary Material

Supplementary Table

## Figures and Tables

**Table 1:** Substance use disorder patients participating in psychotherapy incorporating equines age, sex, drug of choice, and total length of stay at the rehabilitation facility.

Age	N/A	34	26	28	24	60
Sex	M	M	M	F	M	M
Drug of Choice	N/A	Opioid, Marijuana	Methamphe-tamine, Cocaine	Methamphe-tamine, Opioid, Cocaine, Marijuana	Methamphe-tamine, Opioid	Alcohol
Length of Stay	N/A	65	36	35	179	30

N/A indicates information was not available for participant; M refers to male, F refers to female.

**Table 2:** Demographics of horses participating in psychotherapy incorporating equines at a substance abuse disorder residential treatment facility.

Sex	Age	Breed	Weight	Height
Mare	10	Mustang	372.85 kg	145 cm
Mare	18	Quarter Horse	449.96 kg	146 cm
Gelding	12	Appaloosa	340.19 kg	134 cm
Gelding	15	Grade Pony	190.51 kg	120 cm

**Table 3:** Change in means (SD) of human neurotransmitter concentrations (pg/μl) pre and post PIE and between weeks of PIE during a substance abuse disorder residential treatment program.

	Week (Average Across Time Points)		Treatment (Average Across Weeks)		P-Value	
	Week 1	Week 2	Pre	Post	Week	Treatment
DA	0.412 ± 0.25 *	-	0.68 ± 0.28	0.15 ± 0.05	-	0.18
HVA	5.34 ± 4.54	0.72 ± 0.11	4.64 ± 4.82	2.19 ± 1.60	**0.03**	0.72
5-HIAA	4.1 ± 0.58	3.83 ± 0.46	4.08 ± 0.50	3.91 ± 0.58	0.95	0.65
5-HT	0.66 ± 0.41 *	-	1.19 ± 0.53	0.12 ± 0.02	-	**0.04**
DOPAC	1.32 ± 0.77	0.14 ± 0.07	1.12 ± 0.84	0.53 ± 0.29	0.76	0.88
Glu	3595.73 ± 1480.68	982.71 ± 222.37	2503.09 ± 1243.71	2510.86 ± 1301.16	0.94	0.79
GABA	308.55 ± 204.94	45.39 ± 11.26	273.27 ± 219.55	124.53 ± 75.18	< 0.01	0.75
3-MT	16.82 ± 10.56	54.59 ± 41.37	70.82 ± 45.99	4.79 ± 0.35	0.04	< 0.01
Arg	404.35 ± 158.71	380.93 ± 44.18	412.17 ± 144.48	370.51 ± 58.04	0.54	0.85
Lys	1449.81 ± 272.26	1874.79 ± 508.13	1564.66 ± 403.77	1807.16 ± 519.15	0.77	0.62
Taurine	459.94 ± 137.04	406.14 ± 73.77	402.33 ± 117.38	457.78 ± 91.97	0.08	0.19

Bolded *P*-values indicate significance at *P* ≤ 0.05;

* Indicates analysis was only conducted on participants (n = 3) during week 1 session of PIE.

**Table 4:** Change in means (SD) of horse neurotransmitter concentrations (pg/μl) pre and post PIE and between weeks of PIE during a substance abuse disorder residential treatment program.

	Week (Average Across Time Points)		Treatment (Average Across Weeks)		P-Value	

	Week 1	Week 2	Pre	Post	Week	Treatment
DA	4.45 ± 6.12*	–	0.18 ± 0.16	8.73 ± 8.68	–	0.31
HVA	5.85 ± 2.28	1.49 ± 0.35	4.49 ± 2.07	3.09 ± 1.69	0.15	0.47
5-HIAA	3.87 ± 0.67	1.63 ± 0.29	3.11 ± 0.69	2.51 ± 0.70	0.18	0.46
5-HT	0.5 ± 0.43*	–	0.05 ± 0.02	0.95 ± 0.61	–	0.02
DOPAC	0.82 ± 0.44	0.2 ± 0.12	0.48 ± 0.19	0.56 ± 45	0.74	0.72
Glu	10661.64 ± 6191.13	2699.49 ± 506.75	4754.93 ± 1562.45	8777.48 ± 6360.02	0.26	0.48
GABA	2468.21 ± 1460.50	628.16 ± 296.08	1147.94 ± 420.94	1991.74 ± 1511.35	0.99	0.96
3-MT	65.29 ± 43.20	28.07 ± 20.00	53.94 ± 37.64	29.88 ± 22.10	**0.03**	0.01
Arg	222.03 ± 42.78	340.79 ± 71.00	306.15 ± 71.38	292.4 ± 64.11	0.29	0.97
Lys	222.66 ± 73.81	1038.82 ± 369.64	449.29 ± 142.83	1018.75 ± 426.16	0.01	0.42
Taurine	224.64 ± 46.78	693.48 ± 174.09	443.58 ± 129.56	603.06 ± 200.25	< 0.01	**0.03**

Bolded *P*-values indicate significance at *P* ≤ 0.05;

* Indicates analysis was only conducted on participants (n = 3) during week 1 session of PIE.

**Table 5:** Correlations between human and horse neurotransmitter concentrations (pg/μl) pre PIE during a substance abuse disorder residential treatment program.

	Average Neurotransmitter Concentration Prior to Treatment		Correlation Coefficient	P-Value

	Human	Horse		
DA	0.68 ± 0.28	0.18 ± 0.16	0.75	0.53
HVA	4.64 ± 4.82	4.49 ± 2.07	0.20	0.53
5-HIAA	4.08 ± 0.50	3.11 ± 0.69	0.65	**0.02**
5-HT	1.19 ± 0.53	0.05 ± 0.02	0.75	0.46
DOPAC	1.12 ± 0.84	0.48 ± 0.19	0.43	0.16
Glu	2503.09 ± 1243.71	4754.93 ± 1562.45	0.16	0.61
GABA	273.27 ± 219.55	1147.94 ± 420.94	0.17	0.59
3-MT	70.82 ± 45.99	53.94 ± 37.64	−0.32	0.48
Arg	412.17 ± 144.48	306.15 ± 71.38	−0.48	0.27
Lys	1564.66 ± 403.77	449.29 ± 142.83	−0.28	0.55
Taurine	402.33 ± 117.38	443.58 ± 129.56	0.44	0.32

Bolded *P*-values indicate significance at *P* ≤ 0.05

**Table 6:** Correlations between human and horse neurotransmitter concentrations (pg/μl) post PIE during a substance abuse disorder residential treatment program.

	Average Neurotransmitter Concentration Following Treatment		Correlation Coefficient	P-Value

	Human	Horse		
DA	0.15 ± 0.05	8.73 ± 8.68	0.20	**< 0.01**
HVA	2.19 ± 1.60	3.09 ± 1.69	0.43	0.16
5-HIAA	3.91 ± 0.58	2.51 ± 0.70	−0.17	0.60
5-HT	0.12 ± 0.02	0.95 ± 0.61	0.99	**0.05**
DOPAC	0.53 ± 0.29	0.56 ± 45	0.70	**0.01**
Glu	2510.86 ± 1301.16	8777.48 ± 6360.02	0.66	**0.02**
GABA	124.53 ± 75.18	1991.74 ± 1511.35	0.45	0.14
3-MT	4.79 ± 0.35	29.88 ± 22.10	0.40	0.38
Arg	370.51 ± 58.04	292.4 ± 64.11	0.31	0.50
Lys	1807.16 ± 519.15	1018.75 ± 426.16	−0.04	0.93
Taurine	457.78 ± 91.97	603.06 ± 200.25	0.60	0.15

Bolded *P*-values indicate significance at *P* ≤ 0.05

## Appendix A. Supporting information

Supplementary data associated with this article can be found in the online version at doi:10.1016/j.eqre.2025.100035.
