# Supplementary material for: Substance withdrawal during psychotherapy incorporating equines: A preliminary investigation of the role of neurotransmitters during treatment
Source: J Equine Rehabil. Author manuscript; Available in PMC 2025 Oct 6. (PMC12497389; doi:10.1016/j.eqre.2025.100035)
Supplement: Supplementary Table [file NIHMS2112889-supplement-Supplementary_Table.docx]

**Supplemental Materials**

Table S1 Psychotherapy incorporating equines session themes and activities.

| **Theme:** | **Activities:** |
| --- | --- |
| Separation Anxiety | Separate two herd-bound horses and observe behavior of both. Related to strains put on addicts and their families |
| Perspective | Attempt to halter horses using limitations (e.g., bungee cords). Discuss predator and prey responses, perspectives, and how these impact behavior |
| Extended Appendages | Link arms and try to saddle horse with two linked participants (“right brain” and “left brain”) directing two other linked participants (“right arm” and “left arm”). Cannot direct those not within their control. Discuss breakdown between brain and body |
| Grounding | Attempt to ground tie three horses. Discuss differences in training levels and how they relate to the effort and stages of recovery |
| Safe Spaces | Build representation of safe space using familiar and unfamiliar objects to horses. Move horse into/through each obstacle with the horse representing addiction and obstacles representing triggers. Discuss how to deal with addiction triggers in life |
| Life’s Challenges | Move horse around, through, and over obstacle course. Discuss the perspective of how problems must be overcome and alternative methods to face them. |
| Respect, Vulnerability, and Clarity | Exploring natural horsemanship tactics, patients work with horses to establish a bond with horses using clear language, respect, and learn to demonstrate vulnerabilities through leading, lunging, and reading body language |
| Types of Trauma | Using horses as examples of different types of trauma. Horses with small doses of trauma throughout their lives are lunged to demonstrate how “little t” trauma presents itself in addicts. Horses with intense trauma are lunged to demonstrate how “big t” trauma presents itself in addicts. Discuss how horses are worked to overcome this and how this can extend to addicts. |
